# Protein arginine methyltransferase 1 may be involved in pregnane x receptor-activated overexpression of multidrug resistance 1 gene during acquired multidrug resistant

**DOI:** 10.18632/oncotarget.7752

**Published:** 2016-02-26

**Authors:** Tingting Li, Ah-Ng Tony Kong, Zhiqiang Ma, Haiyan Liu, Pinghua Liu, Yu Xiao, Xuehua Jiang, Ling Wang

**Affiliations:** ^1^ Key Laboratory of Drug Targeting and Drug Delivery System of Ministry of Education, West China School of Pharmacy, Sichuan University, Chengdu, Sichuan 610041, China; ^2^ People's Hospital of Xishuangbanna Dai Autonomous Prefecture, Jinghong, Yunnan 666100, China; ^3^ Department of Pharmaceutics & Ernest Mario School of Pharmacy, Rutgers, The State University of New Jersey, Piscataway, New Jersey 08854, USA; ^4^ State Drug Clinical Trial Agency, Sichuan Provincial People's Hospital, Sichuan Academy of Medical Science, Chengdu, Sichuan 610065, China

**Keywords:** multidrug resistance, p-glycoprotein, protein arginine methyl transferase 1, pregnane X receptor

## Abstract

**Purpose:**

Pregnane x receptor (PXR) - activated overexpression of the multidrug resistance 1 (MDR1) gene is an important way for tumor cells to acquire drug resistance. However, the detailed mechanism still remains unclear. In the present study, we aimed to investigate whether protein arginine methyl transferase 1(PRMT1) is involved in PXR - activated overexpression of MDR1 during acquired multidrug resistant.

**Experimental Design:**

Arginine methyltransferase inhibitor 1 (AMI-1) was used to pharmacologically block PRMT1 in resistant breast cancer cells (MCF7/adr). The mRNA and protein levels of MDR1 were detected by real-time PCR and western blotting analysis. Immunofluorescence microscopy and co-immunoprecipitation were used to investigate the physical interaction between PXR and PRMT1. Then, 136 candidate compounds were screened for PRMT1 inhibitors. Lastly, luciferase reporter gene and nude mice bearing resistant breast cancer xenografts were adopted to investigate the anti-tumor effect of PRMT1 inhibitors when combined with adriamycin.

**Results:**

AMI-1 significantly suppressed the expression of MDR1 in MCF7/adr cells and increased cells sensitivity of MCF7/adr to adriamycin. Physical interaction between PRMT1 and PXR exists in MCF7/adr cells, which could be disrupted by AMI-1. Those results suggest that PRMT1 may be involved in PXR-activated overexpression of MDR1 in resistant breast cancer cells, and AMI-1 may suppress MDR1 by disrupting the interaction between PRMT1 and PXR. Then, five compounds including rutin, isoquercitrin, salvianolic acid A, naproxen, and felodipline were identified to be PRMT1 inhibitors. Finally, those PRMT1 inhibitors were observed to significantly decrease MDR1 promoter activity *in vitro* and enhance the antitumor effect of adriamycin in nude mice that bearing resistant breast cancer xenografts.

**Conclusions:**

PRMT1 may be an important co-activator of PXR in activating MDR1 gene during acquired resistance, and PRMT1 inhibitor combined with chemotherapy drugs may be a new strategy for overcoming tumor MDR.

## INTRODUCTION

Multidrug resistance (MDR) is a major limiting factor to successful chemotherapy in a variety of cancers [1, 2]. Currently, the acquired high expression of P-glycoprotein (P-gp), the product of the human multidrug resistance gene (MDR1 /ABCB1), is the primary mechanism of tumor MDR [3, 4]. However, the mechanisms by which tumor cells acquire overexpressed P-gp have not been clearly characterized.

Pregnane x receptor (PXR) is an orphan nuclear receptor that have been proven to play an essential role in the development of MDR [5–7]. *In vitro*, tumor cells can be induced to acquire MDR by incubating with anticancer drugs either at low concentrations with persistent exposure or at high concentrations with intermittent exposure [8]. However, pharmacological inhibition or genetic knockdown of PXR attenuates drug-stimulated MDR1 overexpression and reverses drug resistance [9–10]. It is likely that chemotherapeutic agents stimulate MDR1 overexpression in a PXR-activation pathway. According to previous studies [11, 12], the activation of PXR requires a ligand and several co-activators. After the ligand binds to PXR, the co-activators are recruited to PXR to form a PXR-co-activator complex and start the transcription of MDR1. A large number of traditional chemotherapy drugs, such as adriamycin, paclitaxel, microtubule-stabilizing agents, and small molecule tyrosine kinase inhibitors, are exogenous ligands of PXR [13, 14]. It is possible that the binding of these chemotherapy agents and PXR is the initial step for acquired resistance in tumors.

Moreover, epigenetic modifications may affect the activity of PXR [15]. Protein arginine methyltransferase 1 (PRMT1) is an epigenetic modifier that methylates arginine 3 of histone H4 (H4R3) [16]. H4R3 methylation is an early promoter event during gene activation, which is essential for many subsequent histone modifications [17, 18]. Studies suggest that H4R3 methylation facilitates the transcriptional activation of nuclear receptors [19]. PRMT1 functions as a co-activator of the farnesyl X receptor (FXR) and stimulate the transcription of FXR responsive genes through histone H4R3 methylation [20]. Also, PRMT1 have been reported to be a major histone methyltransferase associated with PXR [21, 22]. Hence, PRMT1 may be an important co-activator of PXR in activating MDR1 gene during acquired multidrug resistant.

Arginine methyltransferase inhibitor 1 (AMI-1) is a selective PRMT1 inhibitor [23], which could significantly inhibit the activity of PRMT1. In current study, AMI-1 was used to pharmacologically block PRMT1 in adriamycin-resistant breast cancer cell line MCF7/adr. The mRNA and protein levels of MDR1 were detected by real-time PCR and western blotting analysis. Then, we studied the mechanism by which AMI-1 decrease the expression of MDR1 using immunofluorescence and co-immunoprecipitation. Next, we built a high-throughput drug-screening platform based on fluorescence polarization signal analysis to screen for PRMT1 inhibitors, and 136 candidates were screened. Finally, we used dual luciferase reporter gene systems and nude mice bearing resistant breast cancer cells to identify the anti-tumor effect of PRMT1 inhibitors *in vitro* and *in vivo*.

## RESULTS

### P-gp, PXR, and PRMT1 were highly expressed in resistant breast cancer cells

**Figure 1:**
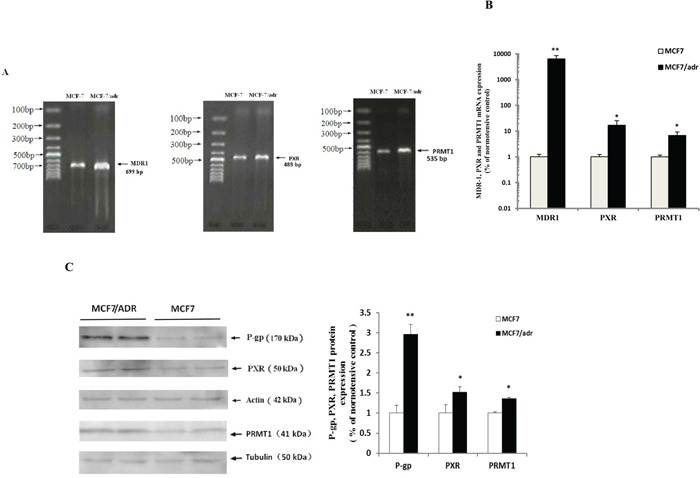
P-gp, PXR and PRMT1 were highly expressed in MCF7/adr cells The expression of MDR1, PXR and PRMT1 mRNA in MCF7/adr and MCF7 cells were examined by **A.** reverse transcription PCR and **B.** Real-time PCR (n=6), using GAPDH as the internal control. **C.** Western blot was used to detect the expression of P-gp, PXR and PRMT1 protein in MCF7/adr and MCF7 cells (n=4). * P < 0.05, ** P < 0.01 compared with MCF7 cells.

Results from reverse transcription PCR (RT-PCR) indicated that MDR1 (699 bp), PXR (489 bp), and PRMT1 (535 bp) mRNA were more highly expressed in MCF7/adr cells than in MCF7 cells (Figure 1a), which were further confirmed by real-time PCR (qPCR) (Figure 1b). Consistently, the protein expression levels of P-gp (170 kDa), PXR (50 kDa), and PRMT1 (41 kDa) were significantly higher in MCF7/adr cells than in MCF7 cells (Figure 1c).

### AMI-1 suppressed the expression of P-gp

**Figure 2:**
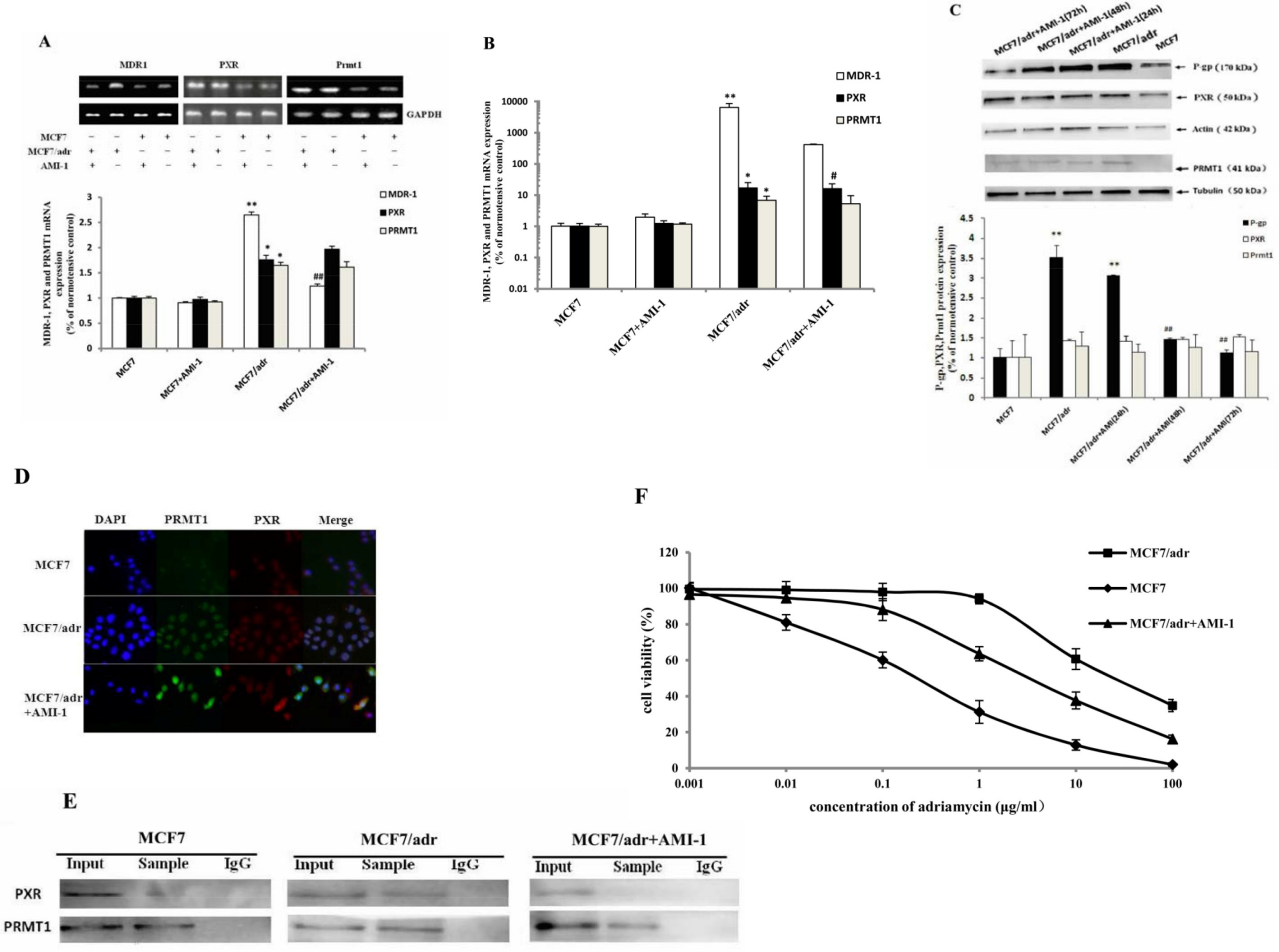
AMI-1 suppresses the expression of MDR1 in MCF7/adr cells The mRNA expression of MDR1, PXR and PRMT1 in MCF7 and MCF7/adr cells after incubating with 4.5 μg/mL of AMI-1 for 6 h were detected by **A.** reverse transcription PCR (n=3) and **B.** real-time PCR (n=6). Protein expression of P-gp, PXR and PRMT1 in MCF7/adr cells after incubating with 4.5 μg/mL of AMI-1 for 24 h, 48 h and 72 h were detected by **C.** western blot (n=3). Effect of AMI-1 (4.5 μg/mL for 24 h) on the **D.** subcellular localization and **E.** interaction between PXR and PRMT1 in MCF7 and MCF7/adr cells. **F.** Cell viability of MCF7, MCF7/adr and AMI-1 pretreated MCF7/adr cells after treating by 0.001~100 μg/mL of Adriamycin (n=3). *, compared with MCF7 cells; #, compared with MCF7/adr cells; * or #, P<0.05; ** or ##, P<0.01.

Based on the possibility that PRMT1 may be an important co-activator of PXR and PXR serves as a pivotal activator of P-gp, we supposed that PRMT1 inhibitor may decrease the expression of P-gp in resistant breast cancer cells. The mRNA and protein levels of P-gp, PXR, and PRMT1 were measured in MCF7/adr and MCF7 cells after incubation with 4.5 μg/mL of AMI-1 [30] for 6 h, 24 h, 48 h, and 72 h. RT-PCR and qPCR results indicated that MDR1 mRNA decreased in MCF7/adr cells after incubation with AMI-1 for 6 h, while MDR1 mRNA in MCF7 cells did not change (Fig. 2a and 2b). Western blotting analysis indicated that P-gp expression declined in a time-dependent manner in MCF7/adr cells after incubation with AMI-1 for 24 h, 48 h, and 72 h (Figure 2c). Moreover, AMI-1 had no significant effect on mRNA expression of PXR and PRMT1 (Fig. 2a and 2b). Likewise, the protein levels of PXR and PRMT1 in MCF7/adr cells were not significantly decreased (Figure 2c). Interestingly, despite being known as a PRMT1 inhibitor, AMI-1 did not significantly decrease the expression of PRMT1. This suggests that the mechanism of AMI-1 may refer to functional inhibition. The subcellular localization of PXR and PRMT1 were highly consistent in MCF7/adr cells, which changed after incubating with 4.5 μg/mL of AMI-1 for 24 h (Figure 2d). This suggests that protein interaction may exist between PXR and PRMT1 within MCF7/adr cells. Next, co-immunoprecipitation was used to test the physical interactions between the two proteins. As shown in Figure 2e, physical interaction between PXR and PRMT1 was detected in MCF7/adr cells, and this interaction could be disrupted by treating the cells with 4.5 μg/mL of AMI-1 for 24 h. These results suggested that the binding of PRMT1 and PXR may be a prerequisite for overexpression of P-gp in resistant cells. Inhibition of PRMT1 suppresses P-gp expression and the mechanism may refer to the disruption of the interaction between PRMT1 and PXR.

It is possible that the sensitivity of resistant cells to antitumor agents increase after incubating with AMI-1 due to the decreased expression of P-gp. To test this possibility, the cytotoxicity of adriamycin (between 0.001 and 100 μg/mL) in MCF7, MCF7/adr, and AMI-1 pretreated MCF7/adr cells were measured. The results showed that IC_50_ decreased approximately 8.6 fold in MCF7/adr cells that were pretreated with 4.5 μg/mL of AMI-1 for 72 h (Figure 2f). This result suggested that inhibition of PRMT1 may increase the sensitivity of resistant cells to antitumor agents and reverse the tumor resistance.

### PRMT1 is required for maintenance of P-gp overexpression

In clinical, once multidrug resistance occurs, resistance continues for a long period of time even if the inducing drugs are removed. Likewise, tumor cells acquire drug resistance by the induction of antitumor agents *in vitro*, and they maintain this resistance for weeks after the inducing drugs are removed [31, 32]. It has been proposed that there are internal MDR1- activating mechanisms in tumor cells that are activated by antitumor agents and continue to act after the agents are removed.

**Figure 3:**
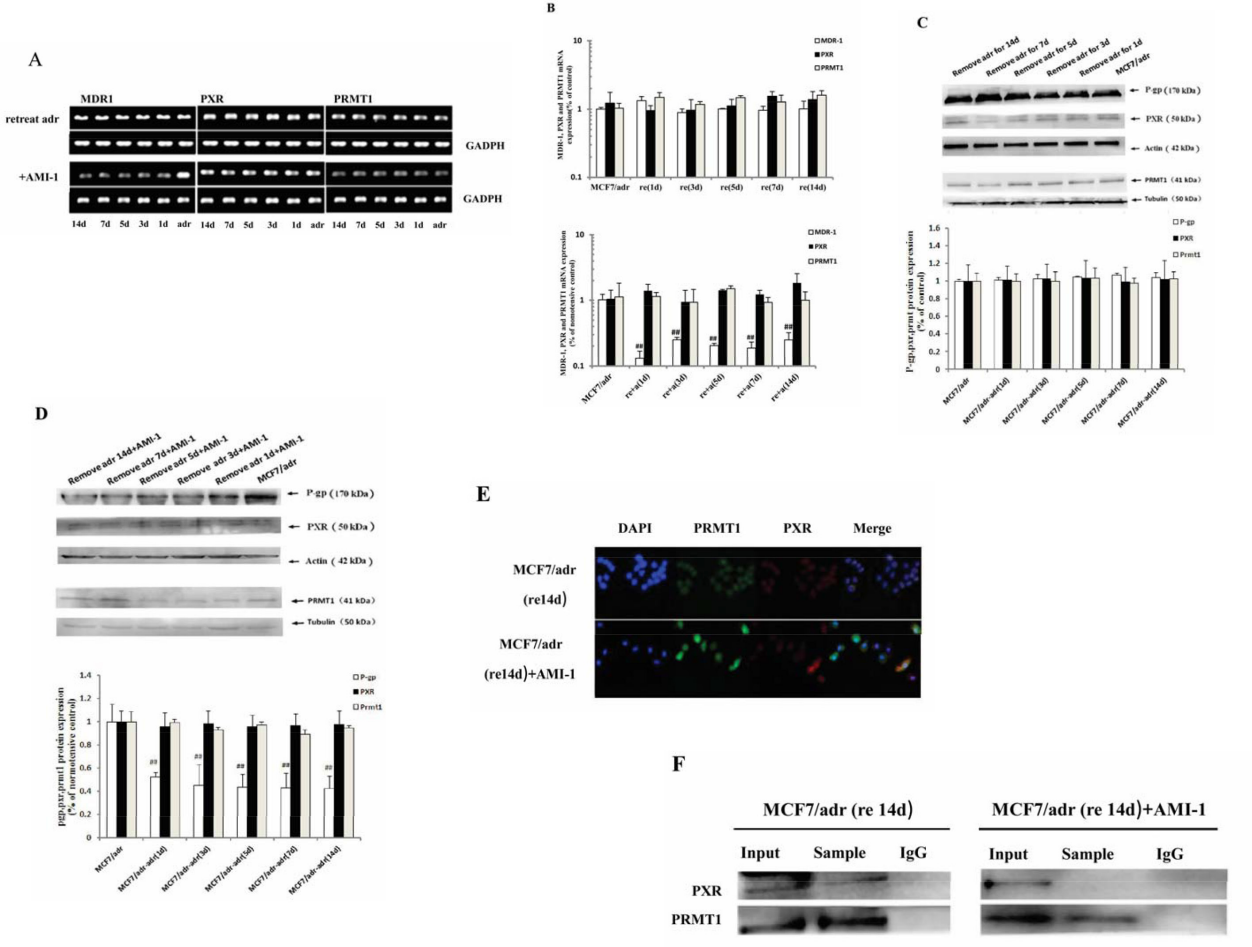
PRMT1 involved in the maintenance of MDR1 overexpression in MCF7/adr cells after removing adriamycin The effect of AMI-1 on mRNA expression of MDR1, PXR and PRMT1 in MCF7/adr cells after removing adriamycin detected by **A.** reverse transcription PCR (n=3) and **B.** real-time PCR (n=6). The **C.** protein expression of P-gp, PXR and PRMT1 in MCF7/adr cells after removing adriamycin and **D.** the effect of AMI-1 on it (n=3). The **E.** subcellular localization and **F.** interaction between PXR and PRMT1 on the 14^th^ days during remove adriamycin experiment. #, compared with MCF7/adr cells; #, P<0.05; ##, P<0.01.

To determine whether PRMT1 is involved in the maintenance of P-gp overexpression after removal of inducing drugs, the expression of P-gp in MCF7/adr cells were monitored for 14 days after removing adriamycin. Since adriamycin was removed from medium 2 weeks before the assay, the first day of experiment was actually the 15^th^ day after removal of adriamycin. As shown in Figs. 3a, 3b and 3c, the mRNA and protein levels of P-gp did not decrease significantly within the 14 days during the experiment, but did appear to decrease after treating with AMI-1 (Figs. 3a, 3b and 3d). The subcellular localization of PRMT1 was still in consistence with PXR on the 14^th^ day of the experiment, and interaction between the two proteins was verified by co-IP (Figures 3e and f). After treatment with 4.5 μg/mL of AMI-1 for 24 h, PXR and PRMT1 partly migrated from the nucleus to the cytoplasm (Figure 3e) and the binding between the two proteins was destroyed (Figure 3f). These results indicated that PRMT1 is required for the maintenance of P-gp overexpression in resistant cells after removal of resistance-inducing drugs.

### Screening of PRMT1 inhibitors

Previous studies have shown that AMI-1 decreased P-gp expression in resistant cells and increased their sensitivity to adriamycin. Hence, PRMT1 seemed to be an effective target for overcoming tumor MDR. In order to identify additional PRMT1 inhibitors, a high-throughput drug-screening platform was developed to screen 136 candidates, including 63 traditional Chinese medicine monomers and 73 chemical drugs.

**Figure 4:**
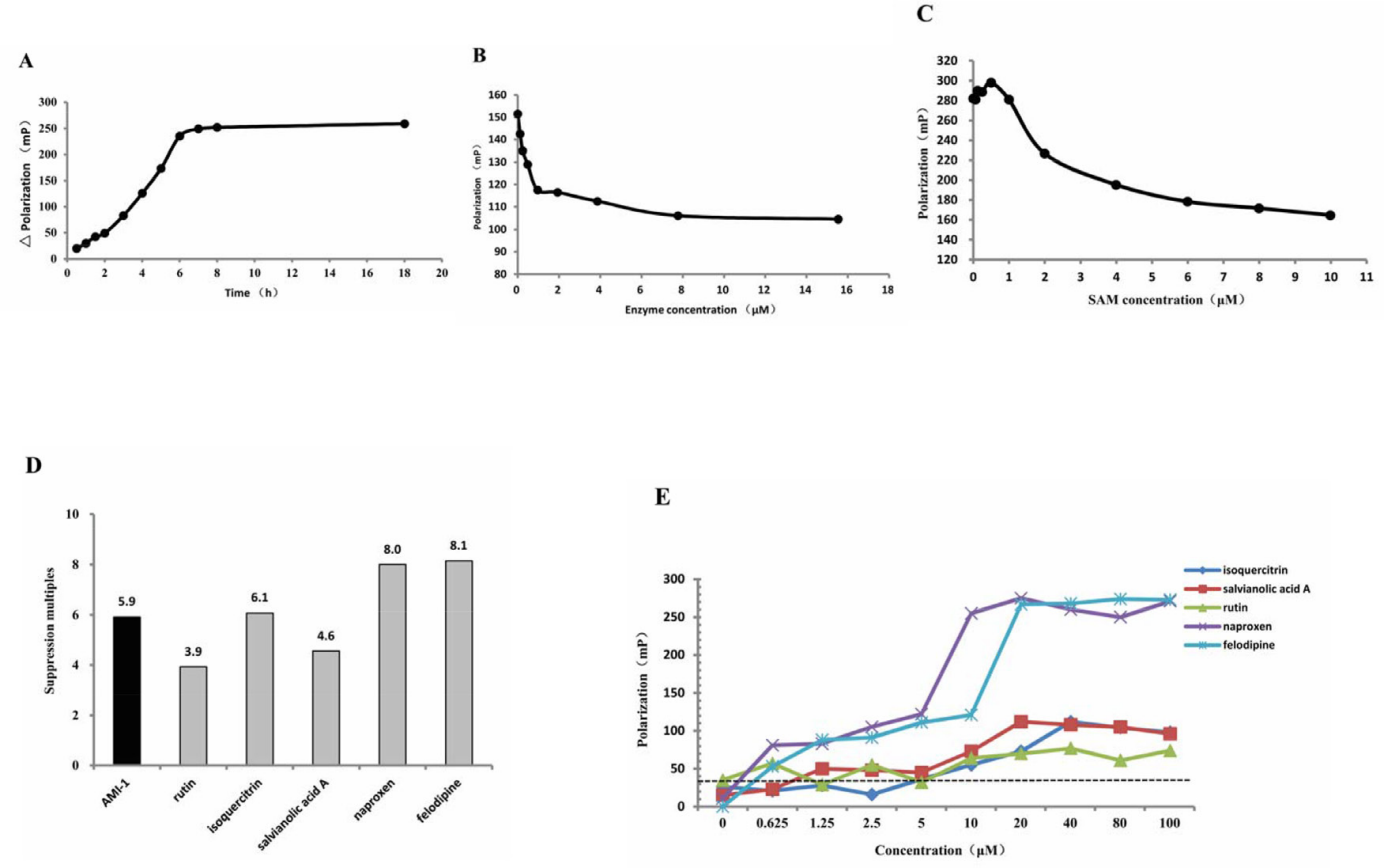
Screening of PRMT1 inhibitors The optimal **A.** incubation time, **B.** PRMT1 enzyme concentration and **C.** SAM concentration of the screening system. **D.** The suppression multiples of AMI-1, naproxen, SAA, rutin, isoquercitrin and felodipline. **E.** The fluorescence polarization (mP) produced by 0~100 μM of naproxen, SAA, rutin, isoquercitrin and felodipline.

Preliminary experiments were carried out to determine the optimal reaction condition, and the results indicated that 6 h, 3.87 ng/μL and 6 μM were the suitable incubation time, PRMT1 enzyme concentration, and SAM concentration, respectively (Figs. 4a, 4b and 4c). Next, the 136 candidates were tested using the screening system. Five compounds were found to inhibit PRMT1 activity, including rutin, isoquercitrin, salvianolic acid A (SAA), naproxen, and felodipline. The suppression multiples of the five compounds were shown in Figure 4d, and the result of rescreening is shown in Fig. 4e. Moreover, AMI-1 significantly inhibited the activity of PRMT1 as expected.

### PRMT1 inhibitors decreased the activity of MDR1 promoter

**Figure 5:**
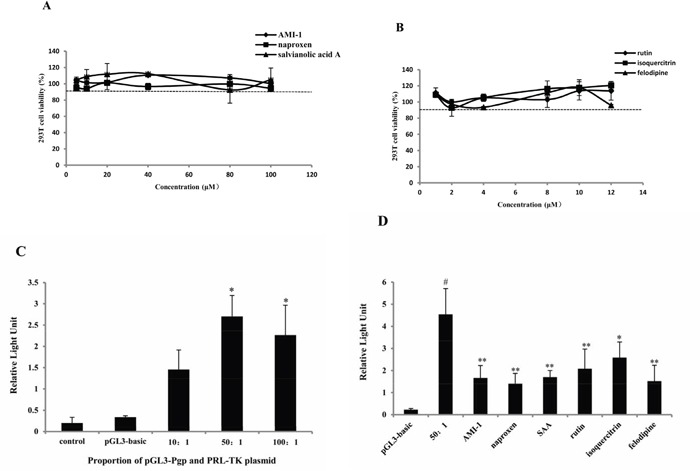
PRMT1 inhibitors decreased the activity of MDR1 promoter Cell viability expressed as a percentage of viable cells compared to control cells after treatment of **A.** AMI-1, naproxen and SAA and **B.** rutin, isoquercitrin and felodipline in HEK 293T cells monitored by the MTT assay (n=3). **C.** Relative light unit caused by pGL3-basic vector and different concentration proportion of pLG3-P-gp and PRL-TK vector by luciferase assay (n=3). **D.** Relative light unit caused by AMI-1 and 10 μM of naproxen, SAA, rutin, isoquercitrin and felodipline (n=3). *, compared with 50:1 group; #, compared with pLG3-basic group; * or #, P<0.05; ** or ##, P<0.01.

The MTT assay [33] in HEK 293T cells indicated that cell viabilities were above 90% when exposed to 5 to 100 μM of AMI-1, naproxen and salvianolic acid A (Figure 5a). However, cell viabilities were significantly increased or decreased when exposure to high concentrations (from 20 to 100 μM) of rutin, isoquercitrin, and felodipline (data not given). The cell viabilities ranged from 90% to 120% when the concentration was reduced to between 1 and 12 μM (Figure 5b). To improve consistency, 10 μM of AMI-1 and the five compounds were used to test the effect on MDR1 promoter activity. As showed in Figure 5c, a 50:1 ratio of pLG3-P-gp to PRL-TK vector seemes to be optimal. The results of luciferase assay indicated that naproxen, SAA, rutin, isoquercitrin, and felodipline all inhibited the promoter activity to different degrees (Figure 5d).

### PRMT1 inhibitors enhanced the antitumor effect of adriamycin in nude mice bearing resistant breast cancer

**Figure 6:**
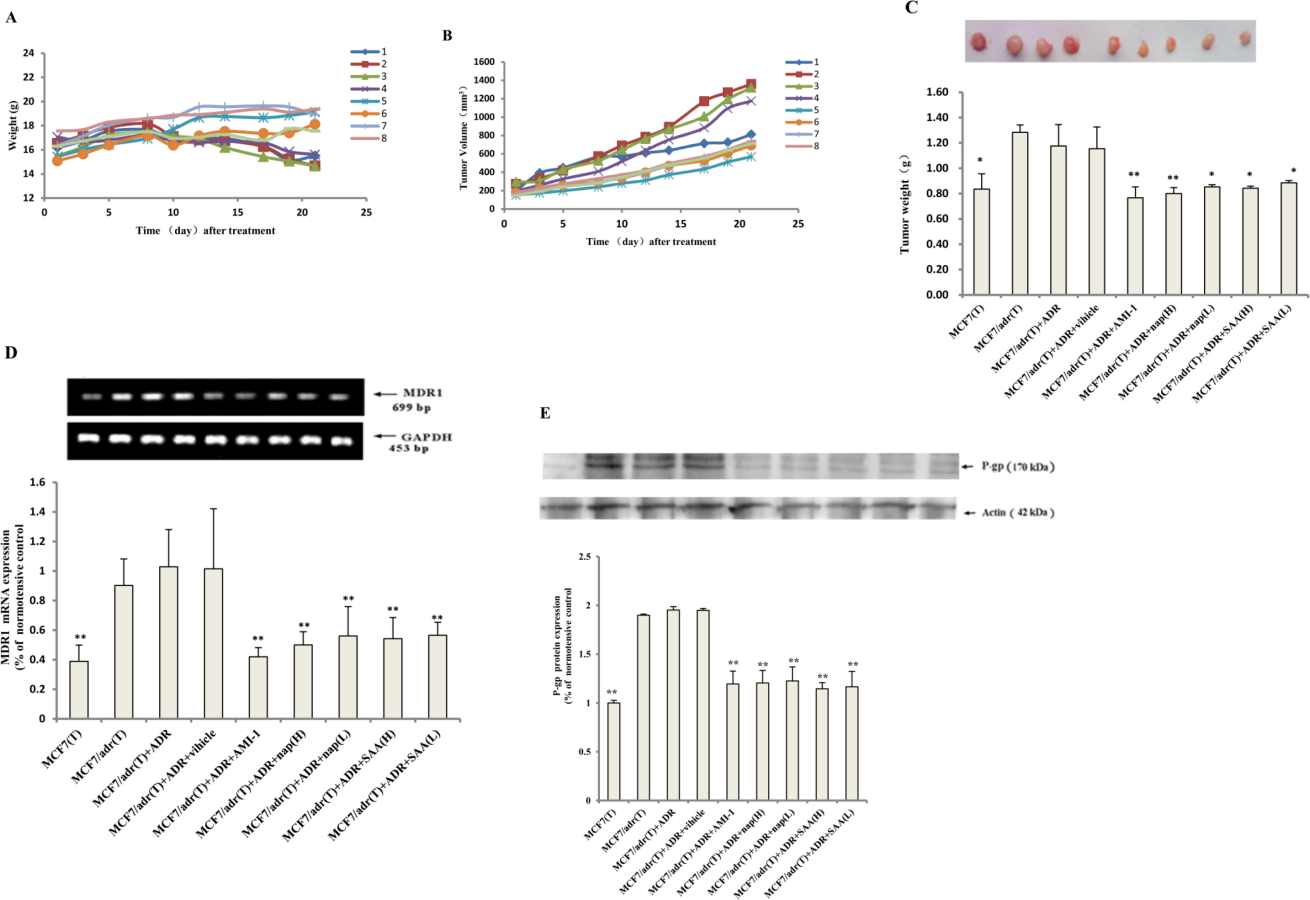
PRMT1 inhibitors enhanced the antitumor effect of adriamycin in nude mice bearing resistant breast cancer The **A.** bodyweight and **B.** tumor sizes of nude mice of the nine groups over time (group 1-9 represent for 1: MCF7 + NS; 2: MCF7/adr + NS; 3: MCF7/adr + adriamycin; 4: MCF7/adr + adriamycin + CMC-Na; 5: MCF7/adr + adriamycin + AMI-1; 6: MCF7/adr + adriamycin + naproxen (H); 7: MCF7/adr + adriamycin + naproxen (L); 8: MCF7/adr + adriamycin + SAA (H); 9: MCF7/adr + adriamycin + SAA (L) respectively, n=3~6). **C.** The tumor weight at the end of the experiment (n=3~6). The MDR1 **D.** mRNA and **E.** protein levels of tumor tissue in each group (n=3). Compared with MCF7/adr+adriamycin (group 3); *, P<0.05; **, P<0.01.

Next, the antitumor effects of naproxen and salvianolic acid A when combined with adriamycin *in vivo* were tested. Compared to administering adriamycin alone, coadministering with naproxen or salvianolic acid A significantly suppressed tumor growth (Figures 6a and 6c) and mitigated the weight loss associated with bearing tumor (Figure 6b). The mRNA of MDR1 in mice treated with both adriamycin and an inhibitor (group 5~9) were significantly lower than that treated with adriamycin alone (group 3) (Figure 6d). Consistently, the protein levels of P-gp were lower in combination therapy groups than monotherapy group (Figure 6e).

## DISCUSSION

As a ligand-dependent nuclear receptor, PXR stimulate gene transcription by directly binding to the DNA after being activated by the appropriate ligand. However, it is difficult for PXR to get the target regions in DNA due to the specific and dense structure of chromosomes. The methylation of histone H4R3, which is catalyzed by PRMT1, is an early promoter event and the beginning of a series of epigenetic modifications during the activation of genes [17]. Previous studies suggest that PRMT1 increases the transcription of PXR responsive gene CYP3A4, and small interfering RNA (siRNA) knockdown or gene deletion of PRMT1 greatly diminishes CYP3A4 expression [34–36]. It is likely that the epigenetic modifications make the dense chromosome structure loose, which helps PXR to arrive at the target regions and facilitates the initiation of transcription. Thus, we hypothesized that PRMT1 acts as a transcriptional co-activator of PXR and plays a role in acquired overexpression of MDR1 in resistant cells. We propose that acquired MDR1 overexpression in tumor cells may be activated by PXR through a tripartite mechanism. First, antineoplastic agents, which serve as exogenous PXR ligands, bind to the PXR and result in allostery of PXR. Then, the PRMT1 binding site on PXR is exposed. Second, PRMT1 is recruited to bind with PXR. PRMT1 methylate histone H4R3 of MDR1 gene, which start the epigenetic modifications and make the chromosome structure loose. Third, PXR-co-activator complex binds to the target region on MDR1 promoter and initiates transcription of MDR1gene.

In the present study, AMI-1 was used to pharmacologically block PRMT1. Our data showed that inhibition of PRMT1 significantly decreased the expression of P-gp in MCF7/adr cells and increased their sensitivity to antitumor agents. The subcellular localization of PRMT1 is highly consistent with PXR in resistant breast cancer cells, and the physical interaction exists between the two proteins. After pharmacologically block PRMT1 by AMI-1, the interaction between PXR and PRMT1 were disrupted, and the expression of P-gp decreased. Hence, we speculate that AMI-1 decrease P-gp expression by disrupting the interaction between PXR and PRMT1. Remarkably, we found that MDR1 overexpression was maintained for at least four weeks after removing adriamycin. Consistently, physical interactions between PXR and PRMT1 still exist on the 28^th^ day after removing adriamycin. Therefore, it is likly that PRMT1 acts as an endogenous agonist of PXR, which maintains transcriptional activity of PXR on MDR1 gene after removing the exogenous PXR ligands. Then, we investigated the effect of PRMT1 inhibitors on P-gp promoter activity *in vitro*, and the result showed P-gp promoter activity were greatly diminished. Also, *in vivo* study indicated that AMI-1, naproxen, and SAA all enhanced the antitumor effect of adriamycin in nude mice bearing resistant breast cancer.

Moreover, we first identified rutin, isoquercitrin, salvianolic acid A, naproxen and felodipline as PRMT1 inhibitors by screening 136 candidates. The result of MTT assay showed that all the five compounds increased the sensitivity of MCF7/adr cells to adriamycin to some extent (supplementary material 4). Among those candidates, 16 traditional Chinese medicine monomers (including glycyrrhetinic acid, tetrandrine, tetramethylpyrazine hydrochloride, ginsenoside Re, ginsenoside Rg1, ginsenoside Rb, panaxadiol, panaxatriol, isoquercitrin, capsaicin, baicalein, quercetin, baicalin, curcumin, piperine and citronella) and 10 chemical drugs (cyclosporine, amiodarone, reserpine, 7-hydroxycoumarin, 7-methyl-coumarin, felodipine, nimodipine, propafenone, verapamil and hymecromone) were previously reported to be P-gp inhibitors [37–45]. However, except for isoquercitrin and felodipine, none of those compounds inhibited PRMT1, which suggests that those 24 compounds may inhibit P-gp though other mechanisms. Both isoquercitrin and rutin are flavonoids and have the same aglycone (supplementary material 5). Interestingly, quercetin, which also has the same aglycone but not glycosylated, did not inhibit PRMT1. In addition, previous studies [46, 47] reported that *in vitro* exposure to salvianolic acid A and naproxen decreased the expression and the efflux function of P-gp though the underling mechanism still unclear. Another study [48] reported that exposure to felodipline decrease the expression of BCRP, which is another PXR responsive gene. It is possible that those drugs suppress the expression of P-gp and BCRP via inhibition of PRMT1.

In conclusion, we have proposed a possibility that PRMT1 may be an important co-activator of PXR in activating MDR1 gene during acquired resistance. Antitumor agents combined with a PRMT1 inhibitor may be an effective strategy for overcoming tumor MDR. However, risks may exist when using PRMT1 inhibitors, since PRMT1 play a crucial role in a wide range of physiological and pathological processes [49–50], and further investigations are needed.

## MATERIALS AND METHODS

### Cell lines and drugs

The human breast cancer cell line, MCF7, and the adriamycin-resistant breast cancer cell line, MCF7/adr, were obtained from Shanghai Institutes for Biological Sciences, Chinese Academy of Sciences (SIBS, CAS, Shanghai, China) and maintained in RPMI 1640 medium (Gibco, New York, NY, USA) at 37°C in a humidified atmosphere of 5% CO_2_. HEK293T cells were supplied by the same institute and were cultured in DMEM medium. RPMI 1640 and DMEM medium were supplemented with 10% fetal bovine serum (HyClone, Logan, UT, USA), 1% nonessential amino acid, 1% glutamine, 1% penicillin and streptomycin. The medium for MCF7/adr cells was additionally supplemented with adriamycin to a final concentration of 1 μg/mL to maintain their resistance. MCF7/adr cells were cultured in drug-free medium for 2 weeks before the assay. Adriamycin was purchased from Sigma Aldrich (Sigma, Louis, MO, USA), and AMI-1 was purchased from Santa Cruz Biotechnology (Santa Cruz Biotechnology, Dallas, TX, USA).

### Reverse transcription PCR and real-time PCR analyses

Total RNA was extracted from cells and tissue using Trizol reagent (Invitrogen, Grand Island, NY, USA). Reverse transcription was performed with 2 μg of total RNA using the RevertAidTM First Strand cDNA Synthesis Kit (Thermo Scientific, Waltham, MA, USA). The resulting cDNA was subsequently amplified using the TakaRa PCR amplification Kit (TakaRa, Kyoto, Japan). The parameters for PCR were as follows: one denaturing cycle at 94°C for 3 min, 35 amplification cycles of 94°C for 30 s, 55°C for 20 s and 72°C for 30 s, one extension cycle of 72°C for 10 min. All products were electrophoresed on 1.5% standard agarose gels that were subsequently stained with ethidium bromide and images were obtained with a gel imaging system (Lingcheng Biological Science and Technology Co Ltd, Shanghai, China).

For real-time PCR, Bestar® SybrGreen qPCR Mastermix (DBI® Bioscience, Ludwigshafen, Germany) was used according to the manufacturer's instructions. Sequences of real-time PCR primers were designed by Primer Express software v 3.0 (Applied Biosystems, Foster City, CA, USA) and were analyzed with BLAST in GenBank database to exclude homology with other genes. The mRNA level was normalized to GAPDH mRNA expression in each sample individually. All primers for reverse transcription PCR and real-time PCR are shown in supplementary material 1.

### Western blotting analysis

Total proteins were extracted from cells and tissue using RIPA Lysis Buffer (Sigma–Aldrich, St. Louis, MO, USA) supplemented with 1% PMSF (Sigma). The concentration of the protein samples was determined and standardized using the BCA Protein Assay Kit (Thermo Scientific, Waltham, MA, USA) according to the manufacturer's instructions. Thirty micrograms of each lysate was electrophoresed by SDS-PAGE on 10% SDS gels, and then were transferred onto a PVDF membrane. After over 1 h of blocking in 5% milk with TBST buffer (20 mM Tris-HCL, 137 mM NaCl, 0.5% Tween 20, pH 7.6), the membrane was incubated with the appropriate primary antibodies of P-gp (Cell Signaling Technology Inc, Beverly, MA, USA), PXR (Abcam, Cambridge, MA, USA) and PRMT1 (Cell Signaling Technology Inc, Beverly, MA, USA) at 4°C overnight. Then, the membrane was washed with TBST buffer for 3 times (10 min each time), and incubated with the corresponding peroxidase-conjugated secondary antibodies (1:2000) for 1 h at 37°C. The membrane was washed again, 3 times with TBST and twice with TBS (10 min each time), and then was exposed to Immobilon Western Chemilum Hrp Substrate (Millipore, Boston, MA, USA). Finally, protein concentration was estimated relative to β-actin (for PXR and P-gp) or tubulin (for PRMT1) using WCIF Image J Software [24].

### Immunofluorescence

Cells were fixed with 4% paraformaldehyde for 10 min and then permeabilized with 0.1% Triton X-100 (Amresco, Solon, OH, USA) for 10 min on ice. After over 3 h of blocking in 10% donkey serum (EY laboratories, San Mateo, CA, USA) with phosphate buffer saline (ZSGB-BIO, Beijing, China), anti-PXR (Abcam, Cambridge, MA, USA) and anti-Prmt1 (Cell Signaling Technology Inc, Beverly, MA, USA) antibodies were added to the blocking solution at a dilution of 1:50 and 1:100, respectively, and incubated overnight at 4°C. Subsequently, cells were incubated with TRITC and FITC tagged secondary antibodies (ZSGB-BIO, Beijing, China) for 1 h at 25°C. After being washed three times (10 min each time), 1 mg/mL of DAPI (Sigma, Louis, MO, USA) was used to stain the nuclear DNA. Finally, cells were analyzed and images were obtained using an inverted fluorescence microscope (Leica, Hessen, Germany).

### Co-immunoprecipitation

Cells were washed twice with PBS and homogenized in the cell lysis buffer for western and IP (Invitrogen, Grand Island, NY, USA). PMSF was added immediately before to the lysis buffer. After centrifugation at 12,000 g for 15 min at 4°C, supernatant fractions were collected and transferred into three tubes prepared as IP sample, negative control, and input control. The supernatant prepared for IP sample was incubated with anti-PRMT1 antibodies (Cell Signaling Technology Inc, Beverly, MA, USA) overnight at 4°C, and then with Protein A + G agarose (Invitrogen, Grand Island, NY, USA) for 2 h at 4°C on a rotary shaker. Corresponding isotype IgG was used instead of anti-PRMT1 antibodies to prepare the supernatant for the negative control. The beads were washed three times and boiled for 10 min with the indicated percentage of loading buffer (Invitrogen, Grand Island, NY, USA), and the supernatant for the input control was also boiled under the same condition. The precipitated protein complexes and the input sample were then analyzed by western blot with anti-PRMT1 and anti-PXR antibodies (Abcam, Cambridge, MA, USA).

### Cytotoxicity assay

A modified MTT assay [25] was used to detect the cytotoxicity of adriamycin in MCF7, MCF7/adr, and AMI-1 pretreated MCF7/adr cells. Cells were seeded in 96-well plates (Corning Incorporated, Corning, NY, USA) at a density of 1×10^4^ cells per well and incubated first for 24 h in RPMI 1640 complete medium (Gibco, New York, NY, USA), then in medium containing 100 – 0.001 μM of adriamycin for another 48 h. Untreated control cells were included. Subsequently, 10 μL of 5 mg/mL of MTT (Sigma, Louis, MO, USA) was added to each well, and the plates were incubated at 37°C for 4 h. Then, the media in each well was discarded and 100 μL of DMSO (Amresco, Solon, OH, USA) was added to solubilize the purple blue formazan. After the incubation at 37°C for10 min, absorbance at 570 nm of the dissolved solutions was measured using a Multiskan MK 3 Microplate Reader (Thermo Scientific, Waltham, MA, USA). All tests and analyses were performed in triplicate. Cell viability was calculated as the percentage of the control value. A graph of percentage of cell viability versus concentrations of adriamycin was plotted and used to calculate the IC_50_.

### Screening of PRMT1 inhibitors

The Transzyme HMT PRMT1 Assay Kit (catalog # 9014, Bellbrook labs, Madison, WI, USA) was used to screen for PRMT1 inhibitors according to the manufacturer's instructions. In black 384-well microplates, 15 μL of the reaction mixture, which consisted of the indicated percentage of HMT PRMT1 enzyme, H4 Peptide (1-20), S-adenosyl-L-methionine (SAM), HMT enzyme buffer, and DEPC-treated H_2_O, plus 2.5 μL of the candidate drug solution (100 μM in DMSO) was added to each well. Then, fluorescence polarization was detected using a Synergy H1 Multi-Mode Reader (BioTek, Winooski, VT, USA). The filters and dichroic mirror used were Ex 620/40 nm, Em 680/30 nm and 660 nm mirror. The reaction system was optimized in a preliminary experiment to determine the optimal concentrations of the PRMT1 enzyme and SAM, and the optimal incubation time. An equal volume of DEPC-treated H_2_O and DMSO were used to replace the candidate drug solution and served as the no inhibitor and vehicle control, respectively. AMI-1 was used as the positive control. The fluorescence polarization produced by the o inhibitor control + 3 standard deviations (SD) was set as screening criteria. The sources of 136 candidates are shown in the supplementary material 2. The suppression multiples were calculated using the fluorescence polarization produced by test drugs divided by the fluorescence polarization produced by the no inhibitor control.

### Plasmid construction and luciferase reporter assay

The MDR1 promoter fragment was amplified by PCR using the following primers: 5′-CCGCTCGAGTATGTTAAAGAATTACTTCATCCCCA-3′ (Forward) and 5′- CCCAAGCTTCTTACCTTTTATCTGGTTGCTTCCTGA-3′ (Reverse). Then, the PCR product was analyzed by agarose gel electrophoresis, and the target product (1278 bp) was extracted and cloned into the pGL3-Basic vector (4818 bp) between the XhoI and Hind III sites. The sequence of the inserted gene fragment can be found at the supplementary material 3. The cloned products were confirmed by final sequencing.

HEK293T cells (SIBS, CAS, Shanghai, China) were simultaneously transfected with pGL3-PGP-Basic plasmid and PRL-TK vector, which contains a gene encoding the Renilla luciferase and served as the control. Lipofectamine 2000 (Invitrogen, Grand Island, NY, USA) was used to transfect the cells according to the manufacturer's protocol. Five hours after transfection, cells were replaced with new medium and treated with 10 mM of AMI-1, naproxen, SAA, rutin, isoquercitrin, or felodipline for 24 h respectively. Cells were then collected and lysed, and luciferase activity was measured and analyzed using the Dual Luciferase Reporter Gene Assay Kit (Beyotime, Shanghai, China) with the Synergy™ HT Multi-Mode Microplate Reader (BioTek, Winooski, VT, USA). The luminescence of cell lysates was used as the blank control. The relative light unit (RLU) was calculated to reflect the activity of the MDR1 promoter using the following formula:

RLU = (L_pGL3-PGP-Basic_ –L_Blank_) / (L_PRL-TK_-L_Blank_)

where L is the luminescence that was detected in each group.

### *In vivo* antitumor study using mice with subcutaneous breast cancer

Female BALB/c nude mice (4 weeks of age) were purchased from the Shuo Da Animal Center (Shuo Da, Chengdu, China) and fed in the Laboratory Animal Center of the West China University of Medical Science (Chengdu, China). All animal experiments were performed in accordance with the Guidelines for the Care and Use of Laboratory Animals and were approved by the Institutional Animal Care and Use Committee of Sichuan University.

The resistant breast cancer model was established by subcutaneous injection of 0.2 mL of MCF7/adr cells at a density of 1×10^7^ /mL into the right upper flanks of BALB/c nude mice. Antitumor activities of naproxen or Salvianolic acid A, combined with adriamycin, were determined in mice bearing the resistant breast cancer. Mice bearing nonresistant breast cancer were obtained by injection of MCF7 cells and served as the control group. The mice were divided into nine groups and subjected to antitumor treatment when the tumor diameter reached 0.5 cm (about 15 days after inoculation). MCF7-bearing mice were treated with saline (0.2 mL, intraperitoneal injection, once every four days). Eight groups of MCF7/adr-bearing mice (n=6 per group) were respectively treated with saline (0.2 mL, intraperitoneal injection, once every four days), adriamycin (8 mg/ kg, intraperitoneal injection, once every four days), adriamycin (as mentioned above) + 0.5% CMC-Na (0.2 mL, intraperitoneal injection, once a day), adriamycin (as mentioned above) + AMI-1 (1.575 mg/kg [26], intraperitoneal injection, once a day), adriamycin (as mentioned above) + naproxen (20 or 5 mg/kg [27], intraperitoneal injection, once a day) or adriamycin (as mentioned above) + Salvianolic acid A (30 or 10 mg/kg [28, 29], intraperitoneal injection, once a day) for 21 consecutive days. Tumor size and mice body weights were measured on days 0, 1, 3, 5, 8, 10, 12, 14, 17, 19, and 21 after treatment. The tumor volume was estimated using the formula:

Volume (V) = 0.5×a×b^2^

where a, b are the shorter and longer diameters of the tumors, respectively.

Twenty-four hours after the last administration, mice were sacrificed by cervical dislocation, and the tumors were stripped. Tumor tissues were collected and stored in liquid nitrogen for future detection of P-gp expression.

### Statistical analysis

Quantitative data were expressed as mean ± SD. Statistical analysis of the data was carried out using the software SPSS (version 10.0, Chicago, IL, USA). Means were compared using one-way analysis of variance (ANOVA) and a Student t-test, and p values < 0.05 were considered statistically significant.

## SUPPLEMENTARY MATERIALS


