# Supplementary material for: Protein arginine methyltransferase 1 may be involved in pregnane x receptor-activated overexpression of multidrug resistance 1 gene during acquired multidrug resistant
Source: Oncotarget. 2016 Feb 26;7(15):20236–48. doi: 10.18632/oncotarget.7752 (PMC4991450; doi:10.18632/oncotarget.7752)
Supplement: Supplementary file 1 [file oncotarget-07-20236-s001.pdf]

SUPPLEMENTARY MATERIALS

Supplementary material 1: Sequences (5'to 3') of primers for reverse transcriptase PCR and real-time PCR

Genes	Sequence (reverse transcriptase PCR)	Sequence (real-time PCR)
Human PXR FWD	ACCACAGTCCATGCCATCAC	GGCATGAAGAAGGAGATGAT
Human PXR REV	TCCACCACCCTGTTGCTGTA	TGGGAGAAGGTAGTGTCAAA
Human PRMT1 FWD	CGCCTCTTGAAGAAGTGTCCTG	TACACGCACTGGAAGCAGA
Human PRMT1 REV	CTCGATGGCCGTCACATACA	GGTTGTTCTTGGCGTTGG
Human Pgp FWD	TGAAACAAAACGACAGAATAGTAAC	GCAGCTGGAAGACAAATACACAAA
Human Pgp REV	AATACTAACAGAACATCCTCAAAGC	CCCCAACATCGTGCACATC
Human GAPDH FWD	ACCACAGTCCATGCCATCAC	GATTTGGCCGTATCGGAC
Human GAPDH REV	TCCACCACCCTGTTGCTGTA	GAAGACGCCAGTAGACTC

PXR , pregnane X receptor; PRMT1, protein arginine methyltransferase 1; Pgp, p-glycoprotein; FWD, forward; REV, reverse.

Supplementary material 2: The promoter fragment of MDR1 gene inserted in pGL3-Basic vector

AAGGTACCAGAATTACTTCATCCCCATGTGAccatctcacctcataatcaaatgaccctaaatccctcactaacctacccctgccctcactaaacttaataataaatgctggtatatccagtgcattgttggcaccgtgggaccagaaggtagtgacccccctggacccagctttcactatcttgtgtgtgtctattatttctcaacctgccgatccgcctaagaacaaagagagagccccgttgcattgcaggctgctggccagatcccacaatacatacacagattccaaaatgcattcttaactcttaaaagattttggttacttatcactattctgtctacttttctgtaattgagaaaagtatttgtcaactcatttttctctctgtgacagctcagtcatttacaaagttttattttatactttactccttccttcaatttgtgctaaaacattgtgaaaattaacatttctttggaacacaactttttcattataaattaaatactgatataattgcaaagtaaacaaatgaatttccataaagctaatttatctttatattttccatacttattacttcaaattcttgttacattttcaattttgtttgaaatatcatatggtattttaaattgaaatttactaattattttttagccagtggataaagagaaattaaaaatttgaacaaattaattcaatttttacttcacttctcatttgaaggtcttcccagtaacctaccaaaagaagtctctctcttttttttttttttttttagcttagattctaatcttcattctgcctattctggctaaacttctcaactctggctatttttcaattataccccaatccctaagccatgtaactcttcgaggtttttgtttgtttttcaactgctcattaagacgtcctacaccttagcaaaaagatcacacatatttcttcaatgctttggagccatagtcatgtactcaaaatttattttatctctactcccacccttcctccacccaaacttatccttgggttttcactgattagtgctttcaagcctgcctgccttagttcatgtagctcctcctctggtactgggataaacacttgtattaccattttaaaggctatcattactctttacctgtgaagagtagaacatgaagaaatctactttattcagatattctccagattcctaaagattagagatcatttctcattctcctaggagtactCACTTCAGGAAGCAACCAGATAAAAGCTTAT.

Supplementary material 3: The candidates for PRMT1 inhibitors Screening and their availability

Drugs	Supplied by
Amiodarone, cyclosporine, verapamil, diphenhydramine, losartan potassium, salicylic acid, paclitaxel, carbamazepine, antipyrine, azelnidipine, omeprazole, tinidazole, cimetidine, butenafine, phenytoin sodium, phenacetin, synephrine, lansoprazole, simvastatin, nateglinide, cefradine, hydrochlorothiazide, acyclovir, gatifloxacin gatifloxacin, ciprofloxacin, uracil, 7-hydroxycoumarin, 7-methyl-coumarin, ephedrine hydrochloride, reserpine, phenformin, benorilate, serotonin hydrochloric acid, naproxen, ganciclovir, probenecid, adenosine, prednisone, chlorpheniramine maleate, valacyclovir hydrochloride, allantoin, acid, mesalamine, Jie losartan, for Misha Tan, alendronate, sulfasalazine, nimodipine, sotalol, propafenone, propranolol, mitoxantrone, tramadol, ranitidine, entecavir, codeine phosphate , chloramphenicol, hymecromone, metoprolol tartrate, ofloxacin, cephalexin, naftopidil, atenolol, Trimethoprim, ketoprofen, clozapine, licorice Acid, tetrandrine, tetramethylpyrazine hydrochloride, ginsenoside Rd, ginsenoside Re, ginsenoside Rh1, ginsenoside Rh2, ginsenosides Rg1, ginsenoside Rb, panaxadiol, Panaxatriol, rutin, isorhamnetin , tectorigenin, tectoridin, tanshinone I, caffeic acid, Tan II a, ursolic acid, baicalein, baicalin, saffron glycosides, Cyclovirobuxine D, borneol, paeoniflorin, psoralen, protocatechuic aldehyde, B- sitosterol, gallic acid, chlorogenic acid, piperine, phillyrin, Vitexin Salidroside astragaloside bat Kudzu base, Helicidum, sennoside A, sennoside B, hesperidin, ferulic acid	National Institute for the Control of Pharmaceutical and Biological Products (Beijing, China)
Rhein, dihydrotanshinone I, psoralen, psoralen, kaempferol glycosides, kaempferol, Salvia acid C, iso Gok hesperidin, capsaicin, licorice acid, paeonol, cryptotanshinone, quercetin, zingerone, glycyrrhizin, isoliquiritin geniposide, curcumin, Sal A, atractylenolide II	Mansite Biotechnology Co., Ltd. (Chengdou,China)
β- estradiol, Furosemide, Isradipine	J&K Scientific Ltd.(Beijing, China)
Citronellal	Tong Wei Industrial Co., Ltd.(Shanghai, China)
Apigenin	You Si Biotechnology Co., Ltd.(Shanghai, China)
Vitexin rhamnoside	Gu Yan Industrial Co., Ltd.(Shanghai, China)
Sulfasalazine	Yu Shi Industrial Co., Ltd.(Guangzhou, China)
Digoxin	Ding Sheng Industrial Co., Ltd.(Henan, China)
Guanidine hydrochloride	Hengrui Pharmaceutical Co., Ltd.(Chengdu, China)

Supplementary material 4

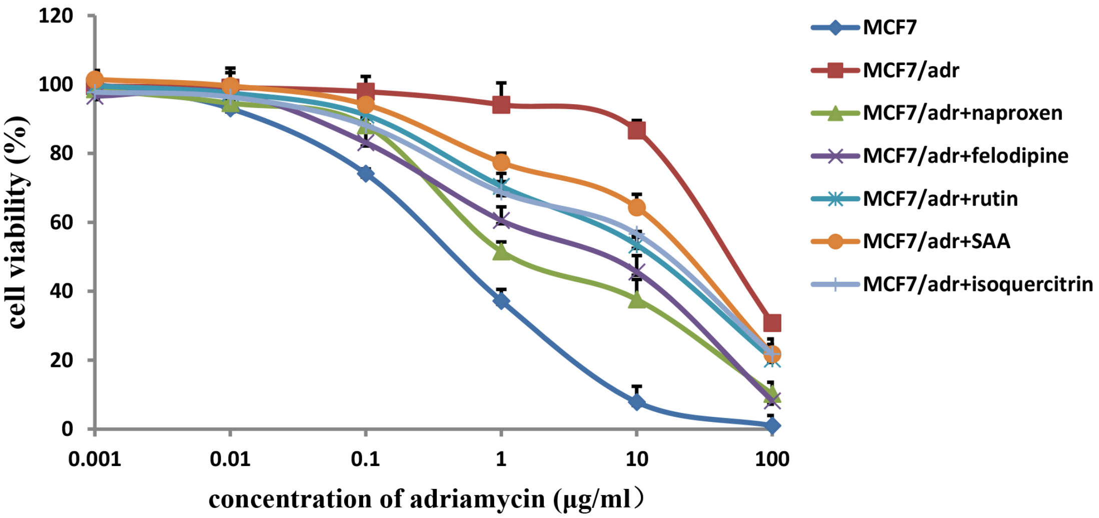

Figure: The effect of naproxen, felodipline, rutin, SAA and isoquercitrin (10μM for 72h) on the sensitivity of MCF7/adr to adriamycin (n=3).

Supplementary material 5

The chemical structure of quercetin, isoquercitrin and rutin

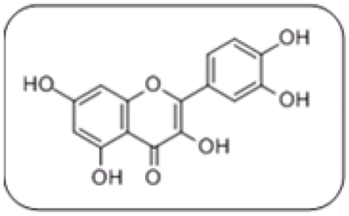

Quercetin

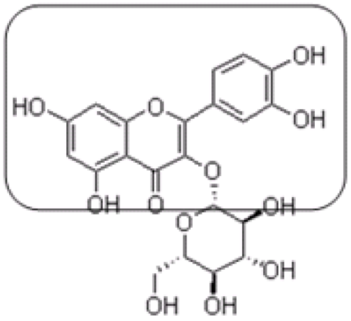

Isoquercitrin

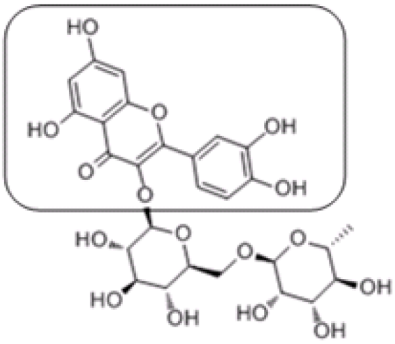

Rutin
